# PYOGENIC LIVER ABSCESS: DIAGNOSTIC AND THERAPEUTIC MANAGEMENT

**DOI:** 10.1590/0102-6720201600030015

**Published:** 2016

**Authors:** Otto Mauro dos SANTOS-ROSA, Henrique Simonsen LUNARDELLI, Marcelo Augusto Fontenelle RIBEIRO-JUNIOR

**Affiliations:** 1 Department of General Surgery, General Hospital of Grajaú, São Paulo, SP, Brazil; 2 Santo Amaro University, São Paulo, SP, Brazil

**Keywords:** Pyogenic liver abscess. Drainage. Ultrasonography, interventional. Hypoalbuminemia.

## Abstract

**Background:**

The pyogenic liver abscess has an incidence of 1.1/1,000 habitants. Mortality can reach 100%. The use of less invasive procedures diminish morbidity and hospital stay.

**Aim:**

Identify risk factors in patients who underwent percutaneous drainage guided by ultrasound as treatment.

**Method:**

Were analyzed 10 patients submitted to the method. Epidemiological characteristics, laboratory markers and imaging exams (ultrasound and CT) were evaluated.

**Results:**

The majority of the patients were men with mean age of 50 years old. Liver disease, alcoholism and biliary tract disease were the most common prodromes. Abdominal pain (90%), fever (70%) and jaundice (40%) were the most common clinical manifestations. Mortality of 20% was observed in this series. Hypoalbuminemia and days of hospitalization had a statistically significant positive association with death.

**Conclusion:**

The pyogenic liver abscess has subacute evolution which makes the diagnosis difficult. Image exams have high sensitivity in diagnosis, particularly computed tomography. Percutaneous drainage associated with antibiotic therapy is safe and effective therapeutic resource.

## RESUMO

**Racional:**

O abscesso hepático piogênico tem incidência de 1,1 por 1.000 habitantes com mortalidade podendo chegar a 100%. O uso de recursos menos invasivos diminuem morbimortalidade e tempo de internação hospitalar.

**Objetivo:**

Identificar fatores de risco no abscesso hepático piogênico tratado por drenagem percutânea guiada por ultrassom.

**Método:**

Total de 10 pacientes foram submetidos ao procedimento. Foram avaliadas características epidemiológicas, marcadores laboratoriais exames de imagem (ultrassom e tomografia).

**Resultados:**

Na amostra houve predominância do sexo masculino, com média de idade de 50 anos. Hepatopatia, etilismo e doença da via biliar foram os pródromos mais frequentes. Dor abdominal (90%), febre (70%) e icterícia (40%) foram manifestações clínicas mais comuns. Houve mortalidade de 20% nesta série. Hipoalbuminemia e dias de internação hospitalar tiveram associação positiva com óbito estatisticamente significante.

**Conclusão:**

O abscesso hepático piogênico tem evolução subaguda o que dificulta o diagnóstico. Exames de imagem têm sensibilidade alta na propedêutica diagnóstica, notadamente a tomografia computadorizada. A drenagem percutânea, associada à antibioticoterapia, mostrou ser recurso terapêutico seguro e eficaz.

## INTRODUCTION

Pyogenic hepatic abscess is caused by the development of intra-hepatic pus collection, secondary to a local inflammatory reaction by bacteria infection in the hepatic parenchyma ^9,16^.

It has an incidence varying from 1,1-2,3 for each 100.000 habitants^3^. The main symptoms are abdominal pain - mainly in the right hypochondrium - fever and hepatomegaly. It has a subacute evolution with symptoms varying between 3-120 days^8^.

The etiology can vary according to region. In Central Europe prevails biliary causes, followed by cryptogenic abscess by S*taphylococcus aureus*, S*treptococcus* and *E. coli*. In Southeast Asia, the most prevalent microorganism is *Klebsiella pneumoniae*. Such differences in the microbiological spectrum have implications in risk and course factors of the disease^3^. Pang *et al.* ^14^ found a higher proportion of cryptogenic causes, followed by biliary and portal (appendicitis, diverticulitis). Also, verified association between microbiology and etiology. *E. coli* is associated with biliary diseases; *Klebsiela* with cryptogenic and *Streptococcus milleri* with portal causes.

It is a very harmful disease, always fatal without treatment. Mortality can vary between 5,6-80%^10^ ^,^ ^13^. A series published in 1938, showed mortality of 60-80%^9^. An expressive downfall on the mortality rate, to 13-18%, was possible because of the effective use of antibiotics after 1980^11^. Another decrease was seen in studies with the use of CT scan and ultrasound. The mortality in studies during and after 1990, remained between 4-10%^15^. The development of interventional radiology, with percutaneous drainage guided by image and minimal invasive surgery, on recent decades, are contributing for the increase of patient's survival^11^.

The treatment to be instituted must consider the cause, the service's experience and the access to diagnostic means and treatment. Antibiotics, interventional radiology, and surgical therapy can be used, combined or as a single therapy. However, the combination of interventional radiology (aspiration or drainage) with antibiotics has shown better results in hospitalization, morbidity, mortality and complications^5^ ^,^ ^13^.

The aim of this study was to identify risk factors of pyogenic liver abscess treated by percutaneous drainage guided by ultrasound.

## METHOD

This is a prospective study in which all patients included were treated in Hospital Geral do Grajaú, São Paulo, SP, Brazil for pyogenic hepatic abscess. Ten patients were included in the period between April and September 2015.

The diagnosis of pyogenic hepatic abscess was confirmed based on clinical characteristics, laboratorial and image exams (ultrasound and tomography). Basic demographic characteristics, prodromes, signs and symptoms, exam results, interventions, complications and outcomes were registered in a standardized protocol.

Fever was defined as a temperature measured in the first 24 h bigger than 37,5^o^ C. All other vital signs were defined by the first set of available observations after presentation. Tachycardia was defined as a cardiac rhythm higher or equal 100 beats per minute, and hypotension as a systolic blood pressure below 90 mmHg. For blood test results, the first available test, in the first 48 h was used for reference. Laboratorial tests reference values were defined by the local laboratory normal values. The abscess size was defined as the largest found diameter. In case of multiple abscesses, was considered the largest abscess diameter. The etiology was a presumptive diagnosis, based on previous treatment history, in the first approach and thorough examination by the team.

The patients with pyogenic abscess suspicion were submitted to large spectrum antibiotics therapy. As protocol instituted by the Hospital Infection Control Commission based on the local bacterial flora, quinolone (ciprofloxacin), associated with a nitroimidazole (metronidazole) were used. The antibiotics were kept in use for 4-6 weeks.

The intervention defined in this study was the percutaneous drainage guided by ultrasound, with maintenance of drainage in situ, applied in a surgical environment, with sedation and local anesthesia. It was used a Pigtail drain with free drainage.

### Statistical analysis 

Was realized using Epi Info 7. The categorical variables in each group were compared with the chi-square (if 20% or more of the expected frequencies were below 5), as appropriated. The continuous variables were compared using the independent t-student sample test, p=<0.05 was considered statistically significant.

## RESULTS

**FIGURE 1:**
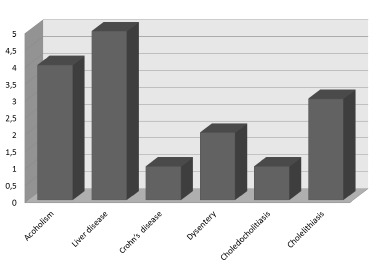
More frequent associated conditions

There was men predominance (70%) with an average age of 50 years old, with peak incidence around 54 years. Half the patients had positive medical history for liver disease. Alcoholism and biliary disease were the most frequent conditions associated (40%). Other diseases were Crohn's and dysentery (Figure 1).

Symptoms duration in average was 10.1 days with peak of 11.5. The patients were hospitalized during 26.2 days in average. Most common symptoms found in admission were abdominal pain 90% (9/10), jaundice 70% (7/10), fever, inappetence and weight loss with 40% (Table 1).

**TABLE 1:** Signs and symptoms

	n (%)
Abdominal pain	9 (90)
Jaundice	7 (70)
Fever	4(40)
Weight loss	4 (40)
Inappetence	4 (40)
Dyspnea	1 (10)
Hypotension	1(10)
Tremors	1(10)
Diarrhea	1(10)
Ascites	2 (20)
Dizziness	2 (20)
Vomiting	1(10)
Dysphagia	1(10)
Asthenia	1(10)
Other	3 (30)
n=patients	

Clinical analysis results in admission are shown in Table 2 with inflammatory alterations (leucocytes and C-reactive protein); 40% had serum levels of urea and creatinine elevated; almost 90% had alterations in hepatic enzymes and hyperbilirubinemia; 90% had altered canalicular enzymes and 75% increased INR.

**TABLE 2:** Clinical analysis results in admission

	Reference	Average±SD	% of patients outside reference value
Leucocytes	05-10 mil/mm^3^	25,62±16,47	89
Hemoglobin	12-15 g/dl	10,99±1,91	0
Platelets	130-450 mil/mm^3^	294,40±134,01	20
PCR	< 5 mg/l	236,10±71,88	100
Urea	18-45 mg/dl	70,50±63,79	44,44
Creatinine	0,7-1,3 mg/dl	2,67±3,61	33,33
Albumin	3,4-4,8 g/dl	2,66±0,69	80
AST	5-34 U/l	204,50±192,75	88,89
ALT	<56 U/l	180,30±149,90	88,89
AP	< 120 U/l	249,11±101,46	100
GGT	12-64 U/l	394,44±291,89	100
Bilirubin	< 1,3 mg/dl	7,5±12,09	75
INR	0,8-1,2	1,46±0,29	75

PCR=C-reactive protein; AST=aspartate transaminase; ALT=aspartate aminotransferase; AP=alkaline phosphatase; GGT=gama glutamic transpeptidase; INR=international normalized relation.

All patients were submitted to ultrasound and tomography and 50% had multiple abscesses with average diameter of 12.54 cm (4-18). Right hepatic lobe was compromised in 90% of the cases and segments VI, VII e VIII in 75% (Table 3).

**TABLE 3:** Univariate analysis of factors that can lead to death

Demographic characteristics and history	Death	Discharge	p-value
Age	52.18 (17-78)	47.3 (37-63)	0.6690
Gender (M-F)	(0-2)	(6-1)	0.0833
Prodrome (days)	8 (0.5-15)	7.6 (2-14)	0.9215
Days in hospital	34 (21-48)	23 (3-34)	0.2680
Clinical signs			
Fever	1	3	0.6666
Abdominal pain	2	7	0.8000
Jaundice	0	7	0.0666
Laboratory			
Leucocytes	24.9 (4.5-67.2)	17.4 (8.8-26.4)	0.5156
Hemoglobin	11.1 (8.5-13.3)	9.1 (7.1-12.2)	0.1048
Platelets	330 (22-555)	343.3 121-468	0.8896
PCR	223.15 ( 96.3-350)	228.48 (158-96.3)	0.5065
Urea	67.4 (29-245)	52.3 (42-63)	0.6875
Creatinine	2.44 (0.6-12.5)	1.1 (0.8-1.6)	0.5513
Albumin	2 (1.6-2.7)	2.8 (2.2-3.6)	0.0402
AST	146.1 (14-433)	221 (5-633)	0.5604
ALT	151.7 (28-509)	124 (6-342)	0.7868
Alkaline Phosphatase	263.2 (136-404)	236 (223-249)	0.7362
GGT	394.4 (137-1121)	113.5 (90-137)	0.2243
Bilirubin	7.7 (0.9-40.1)	1.4 (0.1-2.7)	0.4908
INR	1.38 (1.05-1.9)	1.43 (1.38-1.48)	0.8037
Microbiology			
Positive culture (%)	7 (63.64)	1 (33.33)	0.3846
Image			
Diameter (median)	14 (9.6-18.2)	5 (4-16)	0.0839
Diameter >5cm (%)	1 (12.5)	7(87.5)	0.2222
Right hepatic lobe (%)	2 (22.22)	7 (77.78)	0.8
Left hepatic lobe (%)	0 (0)	1 (12.5)	
Presumed cause			
Cryptogenic	0	2	
Biliary	0	2	
Portal	2	4	

CRP=C-reactive protein; AST=aspartate transaminase; ALT=aspartate aminotransferase; GGT=gama glutamic transpeptidase; INR=international normalized relation

**FIGURE 2:**
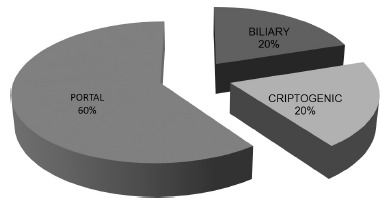
Pyogenic hepatic abscess etiology

The culture was positive in 70% of the patients and the most prevailed microorganism isolated was *Escherichia coli* in 50%, followed by multiresistent beta-lactamase extended-spectrum (ESBL MR) in 25%. Figure 2 shows the etiology.

Every patient received intravenous antibiotic therapy and ultrasound guided drainage; one (10%) received a second drainage; one needed an open surgical approach; two patients died (20%).

## DISCUSSION

Men predominated. Data found in this study is in consonance with others^6^ ^,^ ^10^ ^,^ ^14^. On the present series hepatic abscess was more common in adults with 50 years old, 10 years earlier in relation to literature. The age difference between deaths and good outcome had no statistic significance (p=0.6690). Meddings *et al.* ^10^ in a population based study found patients between 18-84 years old, with 42.9% prevalence between 65-85. Pang *et al.* ^14^ found an average of 64 years and Küster -Filho *et al.* ^8^ average of 30.8 years. In more recent studies, it can be observed a higher preponderance on the elderly.

The clinical presentation show unspecific signals and symptoms. Abdominal pain, jaundice, fever, inappetence and weight loss were the most common. These data are also the ones found in the literature^9^ ^,^ ^14^. This strengthens the fact that hepatic abscess diagnosis is made with high clinical suspicion, given the low prevalence and unspecific symptoms. It can explain the average of 8.2 days for diagnosis, what suggest subacute evolution^14^.

Laboratorial analysis showed leukocytosis (89%, p=0.5156), with an expressive increase in CRP (p=0.5065), average of 236.10 mg/l, although both without statistical significance associated with hypoalbuminemia (p=0.0402) in 80% of patients, suggesting inflammatory response. Unspecific data was also found in other studies^8^ ^,^ ^14^. For Küster-Filho *et al.* ^8^ liver enzymes elevation (AST, ALT), canalicular ones (GGT, AP), and bilirubin elevation are found in higher frequency. These data were not comparable with this series, with these alterations being found respectively in 88.9%; 88.9%; 100%; 100%; and 75% without statistical significance. 

The main cause found in this study was portal, secondary to abdominal cavity contamination. Such results differ from the literature showing preponderance of biliary causes in 44% of patients^8^ or, as Pang et al.^14^, cryptogenic causes in 34%^14^. 

The most common microorganism isolated was *Escherichia coli,* similar to Küster-Filho *et al.* ^8^ and Meddings^10^ papers, despite of etiology. In Brazilian study^8^ biliary causes were more common, but in Americans portal causes^10^ prevailed. Asian series showed a great proportion of patients with *Klebsiella* associated with cryptogenic etiology^4^, mainly in diabetic group^9^.

The abdominal ultrasound had 40% sensibility to diagnose abscesses while abdominal CT scan had 90%. Abscesses were located, in majority, in the right hepatic lobe as single abscesses measuring 12.54 cm. Küster-Filho *et al.* ^8^ found preponderance of multiple abscesses, in the right lobe, measuring 7.33 cm in average.

Antibiotics associated with puncture and drainage have found better results in reference of morbidity and mortality, compared to antibiotic therapy and puncture or open surgery^5^ ^,^ ^8^ ^,^ ^14^. Yu-Long *et al.* ^16^ in a systematic revision with meta-analysis of five RCT comparing puncture and drainage concluded that drainage was superior in success rate (p=0.04), clinical improvement (p=0.0001) and reduction in days to reduce the abscess size in 50% (p<0.00001). 

This study showed mortality of 20% , high when compared to Pang *et al.* ^14^ (6.3%) and a historic Australian series (8%)^1^, but explained by the enrollment of bad cases, one patient with Chron's disease presented generalized peritonitis and multiples enterocutaneous fistulae and another had bronchial aspiration. In the USA this rate is around 5.6%, decreasing from prior 6-19%^10^.

Hospital stay showed association with death, with an average of 48 days in the death group (p=0.0153), while in good outcome it was in average 21.63, not surpassing 34 days.

## CONCLUSION

The pyogenic liver abscess has subacute evolution which makes the diagnosis more difficult. Image exams have high sensitivity, particularly computed tomography. Percutaneous drainage associated with antibiotic therapy is safe and effective therapeutic resource.
